# Comparison the effects and side effects of Covid-19 vaccination in patients with inflammatory bowel disease (IBD): a systematic scoping review

**DOI:** 10.1186/s12876-022-02460-1

**Published:** 2022-08-20

**Authors:** Elham Tabesh, Maryam Soheilipour, Mohammad Rezaeisadrabadi, Elahe Zare-Farashbandi, Razieh Sadat Mousavi-Roknabadi

**Affiliations:** 1 Isfahan Gastroenterology and Hepatology Research Center, Isfahan University of Medical Sciences, Isfahan, Iran; 2 Clinical Informationist Research Group, Health Information Research Center, Isfahan University of Medical Sciences, Isfahan, Iran; 3 Emergency Medicine Research Center, Shiraz University of Medical Sciences, Shiraz, Iran; 4 Health System Research, Vice-Chancellor of Treatment, Shiraz University of Medical Sciences, 5th Floor, Administration Building of Shiraz University of Medical Sciences, Zand St., 71348-14336, Shiraz, Iran

**Keywords:** Side effects, Covid-19 vaccines, Immunity, Inflammatory bowel disease

## Abstract

Covid-19 is a pandemic disease that is more severe and mortal in people with immunodeficiency, such as those with inflammatory bowel disease (IBD). On the other hand, no definitive treatment has been identified for it and the best way to control it is wide spread vaccination. The aim of this study was to evaluate the benefits and side effects of different vaccines in patients with IBD. Three Electronic databases [Medline (accessed from PubMed), Scopus, Science Direct, and Cochrane] were searched systematically without time limit, using MESH terms and the related keywords in English language. We focused on the research studies on the effect and side effects of Covid-19 vaccination in patients with IBD. Articles were excluded if they were not relevant, or were performed on other patients excerpt patients with IBD. Considering the titles and abstracts, unrelated studies were excluded. The full texts of the remained studies were evaluated by authors, independently. Then, the studies' findings were assessed and reported. Finally, after reading the full text of the remained articles, 15 ones included in data extraction. All included studied were research study, and most of them (12/15) had prospective design. Totally, 8/15 studies were performed in single-center settings. In 8/15 studies, patients with IBD were compared with a control group. The results were summarized the in two categories: (1) the effect of vaccination, and (2) side effects. The effect of vaccination were assessed in 13/15 studies. Side effects of Covid-19 vaccination in patients with IBD were reported in 7/15 studies. Patients with IBD can be advised that vaccination may have limited minor side effects, but it can protect them from the serious complications of Covid-19 and its resulting mortality with a high success rate. They should be also mentioned in booster doses.

## Highlights

Studies showed that the risk of developing Covid-19 is more worrying in people with immunocompromised conditions, such as inflammatory bowel disease (IBD). On the other hand, no definitive treatment has been identified for it and the best way to control it is wide spread vaccination. The results of this systematic scoping review revealed that patients with IBD can be advised that vaccination may have limited minor side effects, but it can protect these patients from the serious complications of Covid-19. Also, they should be also mentioned in booster doses.

## Introduction

Covid-19 is a contagious disease which causes numerous deaths throughout the world and known as a pandemic disease without definite treatment. Based on current World Health Organization's statistics, the number of affected population is more than 505 million; and more than 6 million died from the disease [1–3]. Despite scientists' efforts and global vaccination against the disease, new strains of the virus are emerging and spreading like: alpha, beta, gamma, delta, and Omicron, which challenge the treatment [4].

In spite of its virulence pattern, the disease transfer extremely rapid and causes complications such as respiratory distress, cardiac condition and liver failure [2, 5–7]. Furthermore, the risk of developing this disease is more worrying in people with immunocompromised conditions. Such as other communicable infections, it causes concern among gastroenterologists for patients who are affected by inflammatory bowel disease (IBD) [8]. This concern is arising due to statistics that showed more than 6.8 million people worldwide have IBD and this prevalence is increasing [9].

Immunosuppressive therapeutic regimens are the most common treatment for IBD which make the patient more prone to infection**.** Severe pulmonary disease like previously diagnosed pattern including pneumonia and acute respiratory distress syndrome (ARDS) with various imaging findings is the most mortal complication which was characterized by the activation of the inflammatory cascade and an increase in inflammatory factors such as C-reactive protein (CRP) and interleukin [10–12]. Hence, there is a possibility that patients with IBD are more vulnerable to affect with Covid-19 due to immunosuppressive drugs that they have consumed as IBD therapy [8].

According to the growing number of IBD patients, widespread and rapid change of Covid-19 variants, and current challenges on effectiveness of Covid-19 on patients with IBD [13, 14], this study aims to conduct a systematic review on the effectiveness of Covid-19 vaccine and its complications in IBD patients.

## Materials and methods

The current systematic scoping review was performed based on the recommendations of the Preferred Reporting Items for Systematic Reviews and Meta-Analyses extension for Scoping Reviews (PRISMA-ScR) statement [15].

### Data sources

**Fig. 1:**
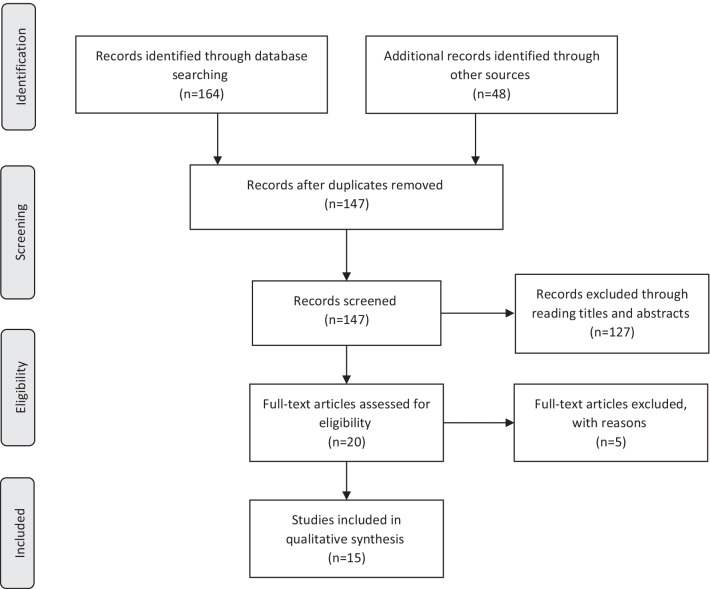
Preferred reporting items for systematic reviews and meta-analyses (PRISMA) flow diagram of the study

As was shown in Fig. 1, a multi-step search strategy was implemented. The electronic literature searches were conducted to identify all relevant studies on Medline (accessed from PubMed), Scopus, Science Direct, and Cochrane without time limit, using MESH terms and the related keywords (Table 1). Google Scholar and researchgate.net were also reviewed manually to explore the grey literature in English. To ensure literature saturation, the reference lists of the included studies or relevant reviews identified through the search were scanned. All the following searches were conducted by two authors [RSM, MR].

**Table 1:** Search strategy used in the present study

*PubMed* (((ulcerative colitis) OR (Crohn's disease)) OR ("Inflammatory bowel disease")) AND (Covid-19 vaccine)Scopus: TITLE(covid OR corona OR sars cov 2) AND TITLE-ABS-KEY(methanol OR alcohol)
*Scopus* ( TITLE-ABS-KEY ( "ulcerative colitis") OR TITLE-ABS-KEY ( "Crohn's disease") OR TITLE-ABS-KEY ( "Inflammatory bowel disease") AND TITLE-ABS-KEY ( "Covid-19 vaccine"))
*Science Direct* "Ulcerative colitis" "Crohn's disease" "Covid-19 vaccine" "Inflammatory bowel disease"
*Cochrane* "ulcerative colitis" "Covid-19 vaccine" "Crohn's disease" "Covid-19 vaccine" "Inflammatory bowel disease" "Covid-19 vaccine"

### Study eligibility criteria

We focused on the research studies on the effect and side effects of Covid-19 vaccination in patients with IBD. Articles were excluded if they were not relevant, or were performed on other patients excerpt patients with IBD, through reading the titles and the abstracts [MR, RSM, ET].

### Participants, and interventions

The target population were all patients with IBD.

### Study appraisal and synthesis methods

Full texts of the studies were evaluated by three authors [MR, ET, RSM]; they decided whether these met the inclusion criteria, independently. They resolved any disagreement through discussions, and finally the articles were selected based on consensus. Neither of the authors were blind to the journal titles or to the study authors or institutions. Then, the level of evidence of each study was determined [16]. The following data were extracted from the included studies and recorded in a Microsoft Excel sheet, 2016: study authors, country, title, methods, sample size, and main findings [MS, EZ, RSM, ET, MR].

### Ethical issues

Ethical issues (including plagiarism, informed consent, misconduct, data fabrication and/or falsification, double publication and/or submission, redundancy, etc.) have been completely observed by the authors.

## Results

In total, 212 (69 articles in Medline, 60 articles in Scopus, 33 article from Science Direct, 2 articles from Cochrane, and 48 articles from other resources) were achieved at the first step search. After initial assessment, 65 duplications were found. After the identification and the screening, 147 articles were selected as potential studies. After reading the full text of these articles, 15 articles formed the final sample and considered for the final data extraction [10, 14, 17–29]. Inter-rater agreement following the first round of screening between the investigators was 85% (Cohen's k = 0.67). Within the second round of screening, inter-rater agreement rose to 100%. Table 2 shows the summary of these studies.

**Table 2:** An overview of studies included in this systematic scoping review and their main findings

Authors (year)	Title	Aim	Sample size	Method	Treatment drugs	Vaccine type	Effects	Side effects	Conclusion	Level of evidence
Botwin et al. [17]	Adverse Events After SARS-CoV-2 mRNA Vaccination Among Patients With Inflammatory Bowel Disease	To evaluate post-mRNA vaccination adverse events in vaccinated adults with IBD patients.	246 (67% CD, 33% indeterminate or UC)	Prospective web-based survey in a longitudinal vaccine registry	Sulfasalazine/mesalamine, budesonide, oral/parenteral Steroids, Mercaptopurine Azathioprine monotherapy, Methotrexate monotherapy, anti- Tumor necrosis factor (TNF) without Mercaptopurine/ Azathioprine/ methotrexate, anti-TNF + Mercaptopurine/ Azathioprine/ Methotrexate, anti-integrin, IL12/23 inhibitor Janus kinase (JAK) inhibitor, Mesalamine	Pfizer, Moderna	Similar to general population More common among younger patients More common in patients with prior Covid-19 Less common in patients receiving biologic therapy Age was associated with side effects after dose 1 (OR = 0.97, *P* = 0.015), suggesting reduced AE risk with each year of advancing age Significant side effects associations after dose 2 included age (OR = 0.97, *P* = 0.018) and biologic status (OR = 0.32, *P* = 0.049), suggesting a reduced side effects risk among biologic recipients, independent of age	Injection-site symptoms, Fatigue/malaise, Headache/dizziness/lightheadedness, fever/chills, Muscle/bone/joint/nerve symptoms, Gastrointestinal symptoms (including nausea, vomiting, diarrhea), Sleep changes, Swollen lymph node, Skin/nail or face changes, Eye/ear/mouth/throat changes, cough, chest/breathing symptoms, memory/ mood changes	IBD and other immune-mediated inflammatory diseases on immunosuppressive and biologic therapies can be reassured that the adverse events risk is likely not increased, and may be reduced, while on biologics.	III
Caldera et al. [14]	Humoral Immunogenicity of mRNA Covid-19 Vaccines Among Patients With Inflammatory Bowel Disease and Healthy Controls	To evaluate humoral immunogenicity of mRNA coronavirus disease 2019 (Covid-19) vaccines among patients with IBD and healthy controls.	182 (122 in IBD group, 60 in control group)	Prospective study	Mesalamine monotherapy, Vedolizumab monotherapy, Thiopurine, Anti-TNF therapy, Anti-TNF combination, Ustekinumab monotherapy or combination, Tofacitinib, Corticosteroid therapy	Moderna, Pfizer	All control group and 97% of patients with IBD developed antibodies Antibody concentrations were lower in patients with IBD Those who received Moderna had higher antibody concentrations compared with those who received the Pfizer vaccine series Patients on immunemodifying therapy had lower antibody concentrations compared with those who were on no treatment, aminosalicylates, or vedolizumab	Not reported	Almost all patients with IBD in our study mounted an antibody response.	II
Cerna et al. [28]	Anti-SARS-CoV-2 Vaccination and Antibody Response in Patients With Inflammatory Bowel Disease on Immune-modifying Therapy: Prospective Single-Tertiary Study	To evaluate the rate and magnitude of seroconversion, assess the effect of different immune-modifying treatment modalities on the magnitude of anti-SARS-CoV-2 IgG antibody levels, and analyze the impact of anti-SARS-CoV-2 vaccination on the inflammatory biomarkers of IBD.	770 (602 in IBD group, 168: control group)	Prospective study	Infliximab, Adalimumab, Vedolizumab, Ustekinumab, Tofacitinib, Thiopurines monotherapy, 5-ASA monotherapy	Pfizer, Moderna, AstraZeneca	The post vaccine seropositivity rate among IBD patients and controls was 97.8% vs 100% Median anti-Covid-19 IgG levels were lower among IBD recipients of AstraZeneca compared with 2 other vaccines and control AstraZeneca recipients No correlation was found between serum trough levels and anti-Covid-19 IgG concentrations for any of the biological drugs used The TNF-α inhibitors with concomitant immunosuppressive treatment but no other treatment modalities were associated with a lower postvaccination antibody response The laboratory activity of IBD evaluated by C-reactive protein and fecal calprotectin levels, and no significant differences were found before the vaccination and 8 weeks after its completion	Not reported	It is necessary to particular attention to the anti-Covid-19 vaccination of IBD patients treated with TNF-α inhibitors with concomitant immunomodulators IBD patients can continue their high-efficacy immune-modifying therapy even during the anti-SARS-CoV-2 vaccination In limited access areas, patients with IBD should be encouraged to receive any readily available vaccine mRNA vaccines are preferred for patients with IBD.	II
Classen et al. [18]	Anti-SARS-CoV-2 Vaccination and Antibody Response in Patients With Inflammatory Bowel Disease on Immune-modifying Therapy: Prospective Single-Tertiary Study	To investigate antibody response to SARS-CoV-2 vaccination in patients with IBD receiving immunomodulators or biologics compared to healthy controls.	144 (72 in IBD group: 55.6% CD and 44.4% UC, and 72 in control group)	Retrospective observational design	Steroids, Mesalazine, Azathioprine, Methothrexate, Calcineurin inhibitor, TNF blocker, Integrin inhibitor, JAK inhibitor, Ustekinumab	Pfizer, Moderna, AstraZeneca	All patients with IBD developed an immune response after full vaccination There was no significant difference in antibody levels between the 3 different vaccines received upon first vaccination Compared to the healthy group, reduced antibody response could be detected There was no vaccination failure in the IBD group after 2 vaccinations There was a trend to a reduced immune response in elderly patients	Muscle pain, Fever, Joint pain, Local redness, Pain injection side, Fatigue, Nausea/vomiting, Diarrhea	A 100% antibody response to vaccination against Covid-19 in patients with IBD and immunomodulatory therapies after 2 vaccinations. Antibody response was high in IBD patients even after the first vaccination – however, antibody levels were lower in IBD patients compared to controls. Overall, vaccination was well tolerated and no further or new adverse events were detected in IBD patients compared to healthy controls.	III
Edelman-Klapper et al. [19]	Lower Serologic Response to Covid-19 mRNA Vaccine in Patients With Inflammatory Bowel Diseases Treated With Anti-TNFalpha	To assess serologic responses to BNT162b2 in patients with IBD stratified according to therapy, compared with healthy controls.	258 (185 in IBD group, 73 in control group) Patients with IBD were divided to 2 separate groups: anti-TNFa group (67) and non-anti-TNFa group (118)	Prospective controlled study	Infliximab, Adalimumab, Vedolizumab, Ustekinumab, 5-ASA, Corticosteroids, Immunomodulatorsc, JAK inhibitor	Pfizer	Covid- anti-S IgG antibodies in all control group were seropositive, whereas about 7% of patients with IBD, regardless of treatment, remained seronegative after dose 1, and it was positive in all patients after dose 2 Anti-TNFa treatment was associated with significantly lower antibody levels Neutralizing and inhibitory functions were both lower in anti-TNFa treated Anti-TNFa drug levels and vaccine responses did not affect anti-spike levels IBD activity was unaffected by vaccination Only anti-TNFa treatment and older age maintained a significant distinct association with lower IgG anti-S response	Local pain, Headache	All patients mounted serologic response to 2 doses of vaccination. Its magnitude was significantly lower in patients treated with anti-TNFa, regardless of administration timing and drug levels. Vaccine was safe. As vaccine serologic response longevity in this group may be limited, vaccine booster dose should be considered.	II
Garrido et al. [20]	"Safety of Covid-19 vaccination in inflammatory bowel disease patients on biologic therapy"	To assess adverse events of Covid-19 vaccination among IBD patients.	239 (76.7% CD and 23.3% UC)	Cohort/ real-life survey: telephone questionnaire	TNF inhibitors, Ustekinumab, Vedolizumab	Pfizer, Moderna, Janssen and AstraZeneca	Not reported	Pain /redness/ Swelling State of sleep/fatigue Headache Myalgia Fever Joint pain Nausea/vomiting Diarrhea Abdominal pain IBD exacerbation	A high acceptance rate and a good safety profile of Covid-19 vaccination in IBD patients treated with biologics Adverse effects were common but overall mild and transitory.	IV
Hadi et al. [21]	Covid-19 Vaccination Is Safe and Effective in Patients With Inflammatory Bowel Disease: Analysis of a Large Multi-institutional Research Network in the United States	To assess safety and efficacy of Covid-19 vaccination in patients with IBD in comparison with the general population without IBD.	864,575, (5562 patients with prior diagnosis of IBD: 2933 UC, 2629 CD)	Retrospective study	Biologics/thiopurines	Pfizer, Moderna	Similar in adverse events of special interest and a new diagnosis of Covid-19 in two groups Similar in the 30-day hospitalization after the Covid-19 vaccination, after matching Similar in steroid prescription at the 1 month follow-up in vaccinated and unvaccinated patients with IBD in unmatched and matched analysis Similar in 30-day adverse events of special interest after the vaccination between patients with IBD with and without biologic or immunomodulator use, and also between patients with CD and UC Similar in steroid use after vaccination was found between patients with and without biologic or immunomodulator use, or both, and between patients with CD and UC	Special adverse events of interest include: acute myocardial infarction, anaphylaxis, facial nerve palsy, coagulopathy, deep vein thrombosis, pulmonary embolism, Guillain-Barré syndrome, transverse myelitis, immune thrombocytopenia, disseminated intravascular coagulation, myocarditis, pericarditis, hemorrhagic stroke, nonhemorrhagic stroke, appendicitis, narcolepsy, and encephalomyelitis	Incidence of Covid-19 in patients with IBD after vaccination is very low, including patients on immunosuppressive agents, and is similar to population without IBD.	III
Kappelman et al. [22]	Humoral Immune Response to Messenger RNA Covid-19 Vaccines Among Patients With Inflammatory Bowel Disease	To assess serologic response after completion of the 2-part mRNA vaccination series in a geographically diverse US IBD population.	317	Prospective study	5ASA, Sulfasalazine, Budesonide, Vedolizumab monotherapy Ustekinumab monotherapy, Mercaptopurine, Azathioprine, Methotrexate, Anti-TNF monotherapy, Anti-TNF combination therapy	Pfizer, Moderna	Antibody response was decreased in IBD patients receiving systemic corticosteroids The proportion of detectible antibodies was 85% among steroid users versus 95% among non-steroid users Antibody response was generally similar across age group, vaccine type, and use of other classes of IBD medications	Not reported	Two doses of mRNA Covid-19 vaccine in a geographically diverse cohort of over 300 patients with IBD, most had detectable antibody responses after the second dose Most patients mount detectable humoral immune response to mRNA vaccinations and support current recommendations to vaccinate patients regardless of immunosuppressive treatment.	IV
Kennedy et al. [23]	Infliximab is associated with attenuated immunogenicity to BNT162b2 and ChAdOx1 nCoV-19 SARS-CoV-2 vaccines in patients with IBD	To investigated whether patients with inflammatory bowel disease treated with infliximab have attenuated serological responses to a single dose of a Covid-19 vaccine.	1293 (Infliximab :865, Vedolizumab-treated patients: 428)	Prospective study	Infliximab, Vedolizumab	Pfizer, AstraZeneca	The concentration of anti-Covid-19 antibody were lower in patients treated with infliximab than vedolizumab, following vaccination Multivariable models showed that antibody concentrations were lower in patients on infliximab compared with vedolizumab Age ≥ 60 years, immunomodulator use, Crohn’s disease and smoking were associated with lower anti-body concentration Non-white ethnicity was associated with higher Covid-19 antibody concentrations Seroconversion rates after a single dose of either vaccine were higher in patients with prior Covid-19 infection and after two doses of Pfizer vaccine	Not reported	Infliximab is associated with attenuated immunogenicity to a single dose of Covid-19 vaccines. Vaccination after Covid-19 infection, or a second dose of vaccine led to seroconversion in most patients. Delayed second dosing should be avoided in patients treated with infliximab.	IV
Lev-Tzion et al. [10]	Covid-19 Vaccine Is Effective in Inflammatory Bowel Disease Patients and Is Not Associated With Disease Exacerbation	To explore the effectiveness of Covid-19 vaccination in IBD and to assess its effect on disease outcomes.	4946	Prospective study	Mesalamine, Corticosteroid, Immunomodulator, Anti-TNF, Vedolizumab, Ustekinumab, Tofacitinib	Pfizer	Overall, 0.3% developed Covid-19 after vaccination (OR = 1) Infection rates were slightly higher in the unvaccinated IBD patients Patients on tumor necrosis factor (TNF) inhibitors and/or corticosteroids did not have a higher incidence of infection No difference in disease outcome was seen during the first 40 days after the second vaccination, however time to flare was shorter in vaccinated compared with unvaccinated IBD patients	The risk of exacerbation was 29% in the vaccinated patients compared with 26% in unvaccinated patients, but it was similar statistically	Covid-19 vaccine effectiveness in IBD patients is comparable with that in non-IBD controls and is not influenced by treatment with TNF inhibitors or corticosteroids. The IBD exacerbation rate did not differ between vaccinated and unvaccinated patients.	III
Levine et al. [29]	COVID-19 Vaccination and Inflammatory Bowel Disease: Desired Antibody Responses, Future Directions, and a Note of Caution	To assess Covid-19 nucleocapsid and spike domain antibodies using a commercially available ELISA assay among consecutively tested postvaccination patients with IBD on biologic or immunomodulator therapy.	19 patients	Prospective study	Biologic therapies: Infliximab, Adalimumab, Golimumab, Ustekinumab, Vedolizumab, Tofacitinib, Methotrexate	Pfizer, Moderna	A 95% overall response rate were observed In patients with elevated spike domain antibodies (a true vaccine response rather than prior undiagnosed infection), 89% (17/19) had the highest measurable levels, at > 250.00 U/mL, with assay reference ranges of 0.79 U/mL indicating negative and 0.80 U/mL (positive results)	Not reported	Time and vaccine availability will lead to the same approach with regard to Covid-19 patients.	IV
Pozdnyakova et al. [24]	Decreased Antibody Responses to Ad26.COV2.S Relative to SARS-CoV-2 mRNA Vaccines in Patients With Inflammatory Bowel Disease	To assess for differences in serologic responses among patients with IBD who received Ad26.CoV2.S relative to those receiving mRNA-1273 or BNT162b2.	353	Prospective study	Immune-modifying therapies (IMTs), as defined by receipt of advanced therapies (biologics or JAK inhibitors), Immunomodulators, and/or systemic Corticosteroids	Moderna, Pfizer, Johnson & Johnson	Two weeks after vaccination, positive antibody levels were detected in more than 90% of IBD patients At week 2, only vaccine type was associated with antibody levels, with both Moderna and Pfizer having significantly higher levels than Jahnson & Jahnson At week 8, vaccine type remained independently associated with antibody levels Lower titers were independently associated with both a longer duration between completion of vaccine regimen and blood sampling and IMT receiving	Not reported	Positive levels of IgG(S) were achieved in virtually all IBD vaccine recipients regardless of vaccine type and IMT use.	IV
Rodriguez-Martino et al. [25]	Early immunologic response to mRNA COVID-19 vaccine in patients receiving biologics and/or immunomodulators	To evaluate humoral and cellular response to Covid-19 vaccines in patients with IBD using biologic and/or immunomodulatory therapies.	19 (CD, 2 UC)	Prospective study	Biologic monotherapy, Azathioprine	Pfizer	Total IgG antibodies increased 21.13 times after dose 1 and 90 times after dose 2 VTN% increased 11.92 times after dose 1 and 53.79 times after dose 2 Total IgG antibodies and VTN% were lower in IBD patients after dose 2 IgG antibodies increased after dose 2, but remained lower than controls VTN% were similar to controls after dose 2 CD4 and CD8 mean levels had an upward trend after vaccinationn	Not reported	Neutralizing capacity response to the vaccine in subjects was similar to a healthy cohort in spite of lower increases in total IgG antibodies. The CD4 and CD8 results observed may support the capacity to mount an effective cellular response in patients on biologics.	IV
Shehab et al. [26]	Serological Response to BNT162b2 and ChAdOx1 nCoV-19 Vaccines in Patients with Inflammatory Bowel Disease on Biologic Therapies	To measure the serological response to BNT162b2 and ChAdOx1 nCoV-19 vaccines in patients with IBD receiving different biologic therapies.	126 (71 CD, 29 UC)	Prospective study	Adalimumab, Infliximab, Vedolizumab, Ustekinumab	Pfizer, AstraZeneca	In patients being treated with infliximab and adalimumab, the proportion of patients who achieved positive anti-Covid-19 IgG antibody levels after receiving two doses of the vaccine were 74.5% and 81.2% In patients receiving ustekinumab and vedolizumab, the proportion of patients who achieved positive anti-Covid-19 IgG antibody levels after receiving two doses of the vaccine were 100% and 92.8% In patients receiving infliximab and adalimumab, the proportion of patients who had positive anti-Covid-19 neutralizing antibody levels after two-dose vaccination were 67.7% and 87.5% The proportion of patients who had positive anti-Covid-19 neutralizing antibody levels were 92.3% and 92.8% in patients receiving ustekinumab and vedolizumab	Not reported	The majority of patients with IBD who were on infliximab, adalimumab, and vedolizumab seroconverted after two doses of Covid-19 vaccination. All patients on ustekinumab seroconverted after two doses of Covid-19 vaccine. The vaccines are likely to be effective after two doses in patients with IBD on biologics.	IV
Wong et al. [27]	Serologic Response to Messenger RNA Coronavirus Disease 2019 Vaccines in Inflammatory Bowel Disease Patients Receiving Biologic Therapies	To evaluated serologic responses to Covid-19 vaccination with Pfizer and Moderna in patients with IBD.	91 (48 in IBD group: 23 CD, 25 UC, 43 in control group)	Prospective study	Infliximab monotherapy, Adalimumab monotherapy, Vedolizumab monotherapy, Vedolizumab plus immunomodulator, Ustekinumab, Tofacitinib, Biologic, anya, Corticosteroids, oralb, Immunomodulatorb, Mesalamineb	Moderna, Pfizer	Side effect was not different in vaccinated IBD patients compared vaccinated non-IBD Anti-TNF were associated with lower anti-RBD total immunoglobulin Vedolizumab was associated with lower anti-RBD total immunoglobulin, anti-RBD IgG, and anti-S IgG than in control group	Local arm pain/swelling/rash, Myalgia, Arthralgia, Fatigue, Headache, Fever/subjective fever, Chills, Gastrointestinal symptoms, Other rash	Results support the consensus recommendation for IBD patients to receive Covid-19 vaccines when available.	IV

Thirteen (13/14) studies were peer-reviewed [10, 14, 17–24, 26–29] and 1/14 of them was in-review article [25]. All included studied were research study, and 12/15 had prospective design [10, 14, 17, 19–24, 26–29] and 4/15 were based on registries [10, 17, 21, 24]. Totally, 8/15 studies were performed in single-center settings [14, 18, 20, 22, 25, 27–29]. In 8/15 studies, patients with IBD were compared with a control group [10, 14, 18, 19, 21, 25, 27, 28].

The studied patients were vaccinated with one of mRNA SARS-CoV-2 such as.

Pfizer (mRNA), Moderna (mRNA), Janseen & AstraZeneca (vector), and AstraZeneca (vector). One study mentioned the most prevalent causes of vaccination refusal in patients with IBD, such as fear of side effects, lack of confidence in the vaccine development process, and little information about vaccination [20]. We summarized the results in two categories: (1) the effect of vaccination, and (2) side effects.

The effect of vaccination were assessed in 13/15 studies [10, 14, 18, 19, 21–24, 26–29]. Measuring antibodies was performed in 10/15 studies [14, 18, 19, 22–24, 26–29]. Side effects of Covid-19 vaccination in patients with IBD were reported in 7/15 studies [17–21, 27, 28]. The mentioned side effects in evaluated articles are presented in Table 3. Localized injection-site were the most common side effect in the studies (5/15) [17–20, 27], following by Fatigue/malaise (4/15) [17, 18, 20, 27] and Myalgia (4/15) [17, 18, 20, 27].

**Table 3:** The reported side effects after Covid-19 vaccination in patients with IBD

Side effects
Localized injection-site [17–20, 27] (5/15)
Fatigue/malaise [17, 18, 20, 27] (4/15)
Myalgia [17, 18, 20, 27] (4/15)
Gastrointestinal symptoms (including nausea, vomiting, diarrhea, abdominal pain) [17, 18, 27] (3/15)
Headache/dizziness/lightheadedness [17, 19, 27] (3/15)
Joint pain [18, 20, 27] (3/15)
Fever/chills [17, 27] (2/15)
IBD exacerbation [20] (2/15)
Skin/nail or face changes [17, 27] (2/15)
Sleep changes [17] (1/15)
Memory/mood changes [17] (1/15)
Swollen lymph node [17] (1/15)
Cough, chest/breathing symptoms [17] (1/15)
Eye/ear/mouth/throat changes [17] (1/15)

## Discussion

In this systematic scoping review, fifteen studies were assessed, which that the obtained results were summarized in two areas. Here, we will discuss the findings.

### The effect of vaccination

Caldera et al. revealed that all control group and 97% of patients with IBD developed antibodies. Antibody concentrations were lower in patients with IBD. Those who received Moderna had higher antibody concentrations compared with those who received the Pfizer vaccine series. Also, patients on immunemodifying therapy had lower antibody concentrations compared with those who were on no treatment, aminosalicylates, or vedolizumab [14].

Also, Cerna et al. stated that the post vaccine seropositivity rate among IBD patients and controls was 97.8% vs 100%. Median anti-Covid-19 IgG levels were lower among IBD recipients of AstraZeneca compared with 2 other vaccines and control AstraZeneca recipients. These were no correlation between serum trough levels and anti-Covid-19 IgG concentrations for any of the biological drugs used. The TNF-α inhibitors with concomitant immunosuppressive treatmen,t but no other treatment modalities were associated with a lower postvaccination antibody response. The laboratory activity of IBD evaluated by C-reactive protein and fecal calprotectin levels. However, there were no significant differences before the vaccination and 8 weeks after its completion [28].

Classen et al. reported that all patients with IBD (100%) developed an immune response after full vaccination. Also, there was no significant difference in antibody levels between the 3 different vaccines received upon first vaccination. The kind of IBD disease and medication had no significant effect on the level of antibody titers. Also, they found that compared to the healthy group, reduced antibody response was detected. There was no vaccination failure in the IBD group after 2 doses vaccinations. In patients with IBD, antibody titers were positively associated with days between last vaccination and blood sample taken, whereas in the control group, antibody titers negatively correlated with the days after dose 1. Moreover, the days between two doses of vaccination had no impact on antibody response in both groups [18].

Similarly, Levin et al. showed a 95% overall response rate after Covid-19 vaccination. Also, none of the patients with positive results for spike domain antibodies had elevations of nucleocapsid antibodies, suggesting a true vaccine response rather than prior undiagnosed infection. In patients with elevated spike domain antibodies (a true vaccine response rather than prior undiagnosed infection), 89% had the highest measurable levels, at > 250.00 U/mL, with assay reference ranges of 0.79 U/mL indicating negative and 0.80 U/mL (positive results) [29].

Lev-Tzion et al. indicated that overall 0.3% developed Covid-19 after vaccination. Infection rates were slightly higher in the unvaccinated IBD patients compare to non IBD patients. Also, patients on tumor necrosis factor (TNF) inhibitors and/or corticosteroids did not have a higher incidence of infection. No difference in disease outcome was observed during the first 40 days after the second vaccination, however time to flare was shorter in vaccinated compared with unvaccinated IBD patients [10].

In another study, Edelman-Klapper et al. found that Covid-19 anti-S IgG antibodies in all control group were seropositive, whereas about 7% of patients with IBD, regardless of treatment, remained seronegative after dose 1, and it was positive in all patients after dose 2. It means that neither IBD itself nor anti-TNFa treatment eliminate the ability to mount serologic response to vaccination. However, anti-TNFa treatment was associated with significantly lower antibody levels compared with non-anti-TNFa treated patients, and control group. Also, neutralizing and inhibitory functions were both lower in anti-TNFa treated compared with non-anti-TNFa treated patients, and control group. Moreover, Anti-TNFa drug levels and vaccine responses did not affect anti-spike levels. But, IBD activity was unaffected by vaccination. The results of multivariate linear regression model showed that only anti-TNFa treatment and older age maintained a significant distinct association with lower IgG anti-S response [19].

Kappelman et al. found antibody response was decreased in IBD patients receiving systemic corticosteroids. In these patients, the proportion of detectible antibodies was 85% versus 95% among non-steroid users. However, antibody response was generally similar across age group, vaccine type, and use of other classes of IBD medications [22].

Moreover, Kennedy et al. showed that the concentration of anti-Covid-19 antibody following vaccination were lower in patients treated with infliximab than vedolizumab. Multivariable models indicated that antibody concentrations were lower in patients treated with infliximab compared with vedolizumab. Age ≥ 60 years, immunomodulator use, Crohn’s disease and smoking were related with lower, while non-white ethnicity was related with higher Covid-19 antibody concentrations. Moreover, seroconversion rates after a single dose of either vaccine were higher in patients with prior Covid-19 infection and after two doses of Pfizer vaccine [23].

In a study by Pozdnyakova et al., it was revealed that two weeks after vaccination, positive antibody levels were detected in more than 90% of IBD patients. Tthe multivariable analysis showed that at week 2, only vaccine type was associated with antibody levels, with both Moderna and Pfizer having significantly higher levels than Jahnson & Jahnson. Also, at week 8, vaccine type remained independently associated with antibody levels. On the other hand, lower titers were independently associated with both a longer duration between completion of vaccine regimen and blood sampling and IMT receiving. They concluded that positive levels of IgG(S) were achieved in virtually all IBD vaccine recipients regardless of vaccine type and IMT use [24].

Furthermore, total IgG antibodies increased 21.13 times after dose 1 and 90 times after dose 2 in Rodriguez-Martino et al.’s study. VTN% increased 11.92 times after dose 1 and 53.79 times after dose 2. Total IgG antibodies and VTN% were lower in IBD patients after dose 2. In their study, IgG antibodies increased after dose 2, but remained lower than control group. However, VTN% were similar to controls after dose 2. CD4 and CD8 mean levels had an upward trend after vaccination [25].

In Shehab et al.’s study, in patients being treated with infliximab and adalimumab, the proportion of patients who achieved positive anti-Covid-19 IgG antibody levels after receiving two doses of the vaccine were 74.5% and 81.2%. Also, it was found that in patients receiving ustekinumab and vedolizumab, the proportion of patients who achieved positive anti-Covid-19 IgG antibody levels after receiving two doses of the vaccine were 100% and 92.8%. In patients receiving infliximab and adalimumab, the proportion of patients who had positive anti-Covid-19 neutralizing antibody levels after two-dose vaccination were 67.7% and 87.5%. The proportion of patients who had positive anti-Covid-19 neutralizing antibody levels were 92.3% and 92.8% in patients receiving ustekinumab and vedolizumab [26].

It was reported in Wong et al.’s study that all IBD patients with 2 doses of vaccination, had positive anti-RBD tests, of whom 84.6% achieved index levels. Also, it was found that anti-TNF were related to lower anti-RBD total immunoglobulin. Moreover, Vedolizumab was associated with lower anti-RBD total immunoglobulin, anti-RBD IgG, and anti-S IgG than in control group. The results of multiple linear regression analyses showed no association between timing of infusion and antibody response [27].

### Side effects

Totally, seven studies mentioned the side effects of Covid-19 vaccinations in patients with IBD [17–21, 27, 28].

In the study by Edelman-Klapper et al., it was reported that immediate and short-term side effects s were detected using phone call and accepted questionnaires, respectively. However, no severe adverse events were reported. Side effects were more after dose 2 compared with dose 1. The most common side effects were local pain (< 70%) and headache (about 30%). Infection rate (about 2%) and side effects were similar in all groups [19].

In another study by Botwin et al., the most common severe symptom after dose 1 was fatigue/malaise (3%); other severe symptoms were reported by 2% or fewer subjects. The most common severe symptoms after dose 2 included fatigue/malaise (10%), fever/chills (8%), and headache (8%). Most symptoms resolved in less than 2 days except for injection site reactions, which mostly resolved within 7 day. Also, it was reported that 39% of patients suffered from side effects after dose 1, and 62% after dose 2. The frequency of side effects was similar to the general population. Also, they found that the frequency of side effects was less common in individuals receiving biologic therapy, and it more in those with prior Covid-19. However, they found that side effects were more common among younger patients, and the massive majority of adverse effects were non-severe. Severe side effects (defined as preventing daily activity) were observed in few patients and 3 patients were hospitalized after dose 1 [17].

Also, Garrido et al. stated that the frequency of side effects was 56.8% after dose 1 and 74.1% after dose 2. Also, it be lower than general population during the first week after vaccination. No serious side effects were reported and all side effects were mild and transitory, and lasted only a few days without any necessity of patients' hospitalization. The percentage of side effects was higher among patients younger than 50 years. However, side effects were reported to be similar in patients with different sex, vaccine type, biological drug or disease type. They finally concluded a high acceptance rate and a good safety profile of Covid-19 vaccination in IBD patients treated with biologics, and diverse effects were common but overall mild and transitory [20].

It was found in Classen et al.’s study that in the IBD group, 58.3% patients had significantly more side effects after dose 1 compared to the control group. But, after dose 2, the side effects were higher in the control group, significantly. The observed side effects after dose 1 were muscle pain, pain at the injection site, and fatigue, which were not significantly higher in IBD patients than in the control group. Similar complaints occurred after dose 2 (with pain at the injection site, fatigue, muscle pain, and fever being the most frequent complaints) [18].

Hadi et al. reported that special adverse events of interest developed in 2.03% patients with IBD, and in 0.81% patients without IBD. There was no significant difference in adverse events of special interest and a new diagnosis of Covid-19 in two groups. Also, it was similar in the 30-day hospitalization after the Covid-19 vaccination, after matching. No difference was found in steroid prescription at the 1 month follow-up in vaccinated and unvaccinated patients with IBD in unmatched and matched analysis. No difference in 30-day adverse events of special interest after the vaccination between patients with IBD with and without biologic or immunomodulator use, and also between patients with CD and UC were found. No difference in steroid use after vaccination was found between patients with and without biologic or immunomodulator use, or both, and between patients with CD and UC [21].

Finally, the results of Wong et al.’s study showed that Covid-19 vaccination’s side effect was not different in vaccinated IBD patients compared vaccinated non-IBD healthcare workers [27].

It is worthy to mention that IBD exacerbation was reported in the Garrido et al. and Lev-Tzion et al.’s studies [10, 20]. IBD exacerbation was defined as treatment escalation, commencement of corticosteroids or enema, or hospitalization. Lev-Tzion et al. found that 44% of vaccinated and 34% of unvaccinated patients experienced an exacerbation or treatment escalation, and this difference was statistically significant. However, the overall risk of exacerbation was 29% in vaccinated patients and 26% in unvaccinated patients, which was statistically similar [10].

Costantino et al. reported a value results on Covid-19 vaccine willingness and hesitancy in Italian IBD patients, as well as the most common reasons. It was mentioned that lack of data on long-term safety can reduce vaccine acceptance. They found that 20% of IBD patients were hesitant or would currently refuse vaccination [30].

The main characteristics of the current systematic scoping review on IBD patients and Covid-19 vaccination was the simultaneous comparison of the complications and benefits of various vaccination. The main limitation of this study was that lack of any clinical trial study, specially randomized controlled trial.

It was concluded that regardless of the vaccine type, IBD patients that receiving immunosuppressive drugs need more careful monitoring of the effects of the vaccine, including screening for antibodies against the Covid-19 virus, as well as more booster doses. On the other hand, the concern that exists among patients with IBD about the side effects of the vaccine was investigated in various studies and it was revealed that the vaccine does not lead to worsening of the disease and the side effects are almost the same like other healthy people. According to existing studies, vaccination has not led to flare of IBD, too.

As a final conclusion, patients with IBD can be advised that vaccination may have limited minor side effects, but it can protect them from the serious complications of Covid-19 disease and its resulting mortality with a high success rate.

## Data Availability

Data sharing is not applicable to this article.
